# Antioxidant Activities of *Dialium indum* L. Fruit and Gas Chromatography-Mass Spectrometry (GC-MS) of the Active Fractions

**DOI:** 10.3390/antiox7110154

**Published:** 2018-11-01

**Authors:** Muhamad Faris Osman, Norazian Mohd Hassan, Alfi Khatib, Siti Marponga Tolos

**Affiliations:** 1 Department of Pharmaceutical Chemistry, Kulliyyah of Pharmacy, International Islamic University Malaysia, Kuantan 25200, Pahang, Malaysia; farisosman@iium.edu.my (M.F.O.); alfikhatib@iium.edu.my (A.K.); 2 Department of Computational and Theoretical Sciences, Kulliyyah of Science, International Islamic University Malaysia, Kuantan 25200, Pahang, Malaysia; smtolos@iium.edu.my

**Keywords:** *Dialium indum*, exocarp, seed, antioxidant, phenolic acids, amino acids, GC-MS analysis

## Abstract

The fruit of *Dialium indum* L. (Fabaceae) is one of the edible wild fruits native to Southeast Asia. The mesocarp is consumed as sweets while the exocarp and seed are regarded as waste. This study aimed to evaluate the antioxidant activities of the fruit by using four assays, which measure its capabilities in reducing phosphomolybdic-phosphotungstic acid reagents, neocuproine, 2,2-diphenyl-picrylhydrazyl (DPPH), and inhibiting linoleic acid peroxidation. The active fractions were then analyzed by gas chromatography-mass spectrometry (GC-MS). The results showed that the seed methanol fraction (SMF) exhibited the strongest antioxidant activity with significantly higher (*p* < 0.05) gallic acid equivalence (GAE), total antioxidant capacity (TAC), and DPPH radical scavenging activity (IC_50_ 31.71; 0.88 µg/mL) than the other fractions. The exocarp dichloromethane fraction (EDF) was the discriminating fraction by having remarkable linoleic acid peroxidation inhibition (IC_50_ 121.43; 2.97 µg/mL). A total of thirty-eight metabolites were detected in derivatized EDF and SMF with distinctive classes of phenolics and amino acids, respectively. Bioautography-guided fractionation of EDF afforded five antioxidant-enriched subfractions with four other detected phenolics. The results revealed the antioxidant properties of *D. indum* fruit, which has potential benefits in pharmaceutical, nutraceutical, and cosmeceutical applications.

## 1. Introduction

Antioxidants of natural origins have been shown to be beneficial in health maintenance and the reduction in the risks of chronic diseases by preventing or removing oxidative damage caused by free radicals [1]. Fruits are among good sources of antioxidants that can be an excellent alternative for the improvement of population health [2]. Wild edible fruits have gained increasing attention worldwide for their good potential to be utilized as functional foods and nutraceuticals owing to their rich content of natural antioxidants and nutrients [3,4]. However, the health promoting benefits of various wild species of edible fruits especially in Asia are still lacking systematic investigation and exploitation when compared with those in Europe and America [5]. Hence, these fruits remain underutilized as natural sources of antioxidants.

Generally, fruit consists of three main parts, namely the mesocarp (pulp), exocarp (skin or peel), and seed. Most studies have been focused on the edible mesocarp, which contains vitamins, minerals, and antioxidant metabolites. Nonetheless, certain fruits accumulate higher levels of antioxidants in their exocarp and seed when compared with the mesocarp. Polyphenols, particularly phenolic acids, and amino acids are among the main antioxidants in the exocarp and seed of fruits [6,7,8,9]. Phenolic acids are one of the most common plant phenolic antioxidants, which possess radical scavenging activities due to their phenolic hydroxyls that enable the compounds to act as reducing agents, hydrogen donors, metal ion chelators, antioxidant enzymes activators, and oxidases inhibitors [10]. On the other hand, amino acids are regarded as synergistic antioxidants, which apart from scavenging radicals also enhance the effects of primary antioxidants through the pro-oxidative chelation of metal traces and regeneration of oxidized primary antioxidants [11].

Located in the Paleotropical Kingdom, Malaysia is endowed with complex tropical rainforest ecosystems, giving rise to about 520 species of plants (trees and non-trees) that produce edible fruits or seeds [12]. *Dialium indum* L. (family Fabaceae), locally known in Malaysia as “keranji”, is a wild tree bearing edible fruit. Synonyms of the plant are *Dialium laurinum* Baker and *Dialium patens* Baker [13]. *D. indum* is native to Southeast Asia as it grows specifically in the forests of Malaysia, Southern Thailand, and Indonesia. The fruit of the plant is also known as black velvet tamarind, derived from its black velvety exocarp and the flavor of the mesocarp that resembles the flesh of the tamarind fruit [14,15].

In Malaysia, the fruit has gained popularity where the name “keranji” is mentioned in a Malay folk poem. To date, only the mesocarp is consumed by locals as sweets, while the seed and the exocarp are discarded as waste. Previous studies on the antioxidant activity of *D. indum* fruit have been conducted specifically on the mesocarp, which showed appreciable in vitro chelation of ferrous ions and scavenging activity against hydroxyls, hydrogen peroxide, 2,2-diphenyl-picrylhydrazyl (DPPH), and nitric oxide radicals. These activities are suggested to be associated with the mesocarp’s total ascorbic acid, β-carotene, lycopene, phenolics, and flavonoids content [16,17].

Studies on the potential health benefits in relation to antioxidant metabolites in this underutilized fruit in the literature have been very scarce. In light of this, a study on the antioxidant content and activities of the fruit is deemed necessary to fill in the research gap. Hence, the objectives of this study were: (i) to determine the antioxidant activities of *D. indum* fruit comprising various extracts and fractions of the exocarp, mesocarp, and seed; (ii) identify the metabolites present in the antioxidant active fractions and subfractions; and (iii) evaluate the correlation of the antioxidant content with the in vitro antioxidant activities.

## 2. Materials and Methods

### 2.1. Chemicals

All reagents were purchased from Merck (Darmstadt, Germany), Sigma–Aldrich (Steinheim, Germany), and Acros Organics (Thermo Fisher Scientific, Reel, Belgium), while all solvents used were of analytical grade.

### 2.2. Plant Materials

Sun-dried *D. indum* fruits were obtained from Bukit Ibam, Muadzam Shah, Pahang, Malaysia and identified by Shamsul Khamis, a botanist from Universiti Kebangsaan Malaysia Herbarium (UKMB). A voucher specimen (PIIUM 0257) was deposited in the Herbarium of Kulliyyah of Pharmacy, International Islamic University of Malaysia, Kuantan. Healthy and uninfected fruits were carefully selected and the exocarp (skin), mesocarp (pulp), and seed of the fruits were separated manually. Each fruit part was further dried in the drying oven (Memmert GmbH + Co. KG, Büchenbach, Germany) at 40 °C for three days, then immediately ground into powder form and kept in plastic containers at room temperature until further use.

### 2.3. Preparation of Extracts and Fractions

One crude extract and three fractions were prepared from each part of the *D. indum* fruit. For the crude extract, 25 g of each fruit part was macerated in 100% methanol (500 mL) at room temperature for 48 h. The extraction procedure was repeated three times. Next, the solvent was removed under reduced pressure using a rotary evaporator (RV 10, IKA^®^, Staufen im Breisgau, Germany) at 40 °C. Using a similar extraction procedure, 75 g of each fruit part was extracted successively with 1.5 L *n*-hexane, dichloromethane (DCM), and methanol to obtain hexane, DCM, and methanol fractions [18]. All extracts and fractions were stored at 4 °C until further use.

### 2.4. Reduction of Phosphomolybdic-Phosphotungstic Acid Reagents

Capability of the extracts and fractions to reduce phosphomolybdic-phosphotungstic acid reagents, otherwise known as Folin–Ciocalteu reagent, was determined according to a previous report with slight modifications [3]. Briefly, 90 µL diluted Folin–Ciocalteu reagent in deionized water (20% *v*/*v*) was placed in each well of a 96-flat-bottomed-well microplate. Then, an 18 µL sample solution in methanol (1000 µg/mL) was added and incubated at room temperature for 5 min, followed by the addition of 90 µL sodium carbonate in deionized water (75 g/L). The mixture was incubated for 2 h at room temperature. The absorbance was then read at 725 nm using a microplate reader (Tecan, Infinite M200 Nanoquant, Männedorf, Switzerland). Gallic acid equivalence (GAE) was determined using a gallic acid calibration curve. All samples were tested in triplicate. Results were expressed as µmol GAE per gram dry weight crude extract or fraction ± standard error of the mean (*SEM*).

### 2.5. Reduction of Neocuproine

The extent of the conversion of light blue-colored bis(neocuproine)copper(II) chelate to yellow-orange-colored bis(neocuproine)copper(I) chelate by antioxidants was measured using the method described by Apak, Güçlü, Özyürek, Bektaşoǧlu, and Bener [19]. An aliquot of 48.8 μL copper(II) chloride in deionized water (10 mM), 48.8 μL neocuproine in 96% ethanol (7.5 mM), and 48.8 μL ammonium acetate buffer (1 M, pH 7.0) were added into wells of a 96-flat-bottomed-well microplate. Then, a 24.4 μL sample solution in 96% ethanol (1000 μg/mL) and 29.3 μL deionized water were added to make up the final volume of 200 µL in each well. The absorbance was measured at 450 nm after 30 min incubation at room temperature. The total antioxidant capacity (TAC) was determined using the standard calibration curve of trolox. All samples were measured in triplicate. Results were expressed as μmol trolox equivalence (TE) per gram dry weight crude extract or fraction ± standard error of the mean (*SEM*).

### 2.6. Scavenging of DPPH Radical

Serial dilution from stock solution of the samples and positive standard (quercetin) in 100% methanol (1000 μg/mL) was done in 30 mL glass vials using a micropipette, then 100 μL of each concentration was transferred to each well of the microplate. A total of 100 μL DPPH in methanol (80 μg/mL) was then added. The mixture was incubated at room temperature for 30 min and the absorbance of the test mixture was then read at 517 nm against the blanks: A_blank sample_ (100 μL of 7.8125–1000 µg/mL sample without DPPH and 100 µL methanol) and A_blank methanol_ (200 μL of 100% methanol). The percentage of inhibition of the DPPH radical was calculated using the equation:

DPPH radical scavenging activity (%) = [1 − (A_control_ − A_sample_)/A_control_] × 100

where A_control_ is the absorbance of DPPH without the sample after subtraction with A_blank methanol_ while A_sample_ is the absorbance of samples with DPPH after subtraction with A_blank methanol_ and A_blank sample_. A_blank sample_ was included in the equation to minimize the effect of varying visible colors of the different extracts to the absorbance readings. For each sample, the assay was conducted in triplicate. The concentration of extracts required to inhibit the DPPH radicals by 50% (IC_50_) was calculated using either the logarithmic or exponential or linear regression model equation that best fitted the data for each sample (*r* > 0.9) [20].

### 2.7. Inhibition of Linoleic Acid Peroxidation

Briefly, 62.5 µL of varying concentrations of the samples (7.8125–1000 µg/mL) and standard (quercetin) were prepared in 1.5 mL Eppendorf tubes. The linoleic acid emulsion was freshly prepared by the emulsification of 100 µL of linoleic acid with 200 µL Tween 20 and 19.7 mL deionized water [21]. Next, 62.5 µL of varying concentrations of the *D. indum* extracts, fractions and standard were mixed with 62.5 µL of the linoleic acid emulsion, 62.5 µL of phosphate buffer (100 µM, pH 7.4), and 12.5 µL of ferrous sulfate solution (4 mM in deionized water). Linoleic acid peroxidation was started by the addition of 12.5 µL ascorbic acid (2 mM in deionized water, freshly prepared for each extract), incubated for 30 min at 37 °C, and terminated by the addition of 187.6 µL of trichloroacetic acid (10% in deionized water). The mixture was then added to 100 µL of thiobarbituric acid solution (1% in 50 mM NaOH), followed by heating in a 100 °C water bath for 10 min. The mixtures were centrifuged at 3500× *g* for 10 min. Then, 100 μL of the supernatant was transferred into the well of a 96-flat-bottomed-well microplate and the absorbance of thiobarbituric acid-reacting substances (TBARS) in the supernatant was measured at 532 nm. For each sample concentration, the assay was conducted in triplicate [22]. The percentage of linoleic acid peroxidation inhibition and IC_50_ were calculated using a similar equation and method described in Section 2.6.

### 2.8. Bioautography-Guided Fractionation

The exocarp dichloromethane fraction (EDF) that exhibited strong antioxidant activities for most assays was further fractionated by column chromatography. A glass column (32 × 1000 mm) packed with silica gel 60 (70–230 mesh, 63–200 μm) was loaded with the fraction at a ratio of 130:1. The column was eluted with binary solvent systems of increasing polarity starting with 200 mL of DCM/hexane (8:2, *v*/*v*) up to 100% DCM at 5% increments to afford five subfractions (Di-1 to Di-5), then DCM/ethyl acetate (EA) (99:1, *v*/*v*) up to the volume ratio of 7:3 at 1%, 2% and 5% increments of EA successively to yield fifteen subfractions (Di-6 to Di-20). Next, DCM/acetone (95:5, *v*/*v* up to 8:2, *v*/*v*) at 5% increments of acetone, yielding two subfractions (Di-21 and Di-22). Finally, DCM/methanol (99:1, *v*/*v*) at 1% and 2% increments of methanol up to 8:2 and 9:1 ratios, respectively, giving four subfractions (Di-23 to Di-26). Antioxidant subfractions were monitored by TLC bioautographic screening using 0.4 mM DPPH in methanol as the spraying reagent and the DPPH radical scavenging activity was evaluated.

### 2.9. Sample Derivatization

Sample derivatization was carried out according to a method described by the manufacturer [23] with modifications. One hundred (100) μL pyridine was added to 1.5 mg of extract in a 1.5 mL Eppendorf tube and the mixture was sonicated at 30 °C for 10 min. Then, 100 μL of silylating agent *N*-methyl-*N*-(trimethylsilyl)trifluoroacetamide (MSTFA) was added, followed by incubation in a water bath at 60 °C for 15 min. Finally, the derivatized sample was syringed out using a 1 mL tuberculin syringe with needle, filtered using a 0.45 μm filter, and injected directly into the gas chromatography-mass spectrometry (GC-MS) instrument.

### 2.10. Metabolite Identification by GC-MS

The derivatized sample was analyzed with an Agilent 6890N Network Gas Chromatography system (Agilent, CA, USA), coupled to an Agilent 5973 Network Mass Selective Detector (Agilent, CA, USA), equipped with an Agilent 7683 Series Injector (Agilent, CA, USA). One µL aliquot of the extract was injected in splitless mode into a DB5-MS + DG column (Agilent, CA, USA) (J&W 122-5532G, 30 m × 250 µm internal diameter low-bleed fused silica capillary column coated with 5% phenyl-95% dimethylarylene siloxane of 0.25 μm thickness, with a built-in 10 m Duraguard column). Helium was used as the carrier gas and the pressure was programmed so that the helium flow was kept constant at a flow rate of 1.2 mL per min. The injector temperature was set at 250 °C.

A series of trials to optimize the column initial and final temperatures, temperature holding times, and temperature increment rates was carried out to obtain the best separation of the chromatogram peaks for EDF (Table S1). The optimized GC parameters were as follows: initial column temperature was isothermal at 100 °C for 12 min, then raised to 140 °C at a rate of 5 °C/min, held for 10 min, and increased to 250 °C at a rate of 5 °C/min. The temperature was then held at 250 °C for 10 min, making a total running time of 62 min. Chromatogram peaks were integrated by using a Chemstation integrator and the mass spectra for each of the peaks were compared with the National Institute of Standards and Technology (NIST) 2014 database library. Since no internal standard was included, only compounds with similarity indices of 90 and above were reported.

### 2.11. Statistical Analysis

Values are mean ± standard error of the mean (*SEM*) of triplicate analysis. Calculation and regression analysis were performed using Microsoft Excel 2016 (Microsoft Corporation, Washington, D.C., USA) and Graph 4.4.2 whereas statistical analysis was carried out using Statistical Package for Social Sciences (SPSS, version 22.0, International Business Machines Corporation, New York, NY, USA). The strength and direction of the association between two ranked variables were measured using the Spearman correlation. The Shapiro–Wilk test was used for assessing the normality of the data distribution while the homogeneity of variances was tested using Levene’s test. Since the assumption of the homogeneity of variances had been violated, multiple comparisons between the various extracts were performed using one-way analysis of variance (ANOVA) of unequal variance (Welch’s ANOVA) with Games Howell post hoc test. Significant difference was accepted at *p* < 0.050 or *p* < 0.010.

## 3. Results

### 3.1. Gallic Acid Equivalence (GAE)

The gallic acid equivalence for the various crude extracts and fractions is shown in Table 1. GAE values varied in the range of 92.97 ± 0.99 (for mesocarp methanol fraction, MMF) to 1405.41 ± 17.96 (for seed methanol fraction, SMF) µmol GAE/g dry extract, using a calibration curve of gallic acid (*r*^2^ = 0.9997). The GAE values were further grouped as the following: low GAE (0–500 µmol GAE/g dry extract), moderate (500–1000 µmol GAE/g dry extract), and high (1000–1500 µmol GAE/g dry extract). All tested extracts and fractions were in the category of low GAE, except for SMF.

### 3.2. Total Antioxidant Capacity (TAC)

Neocuproine reagent confers higher stability when compared with other chromogenic reagents such as 2,2′-azino-bis(3-ethylbenzthiazoline-6-sulfonic acid) (ABTS) and DPPH [20]. TAC for the various *D. indum* fruit extracts (Table 1) varied in the range of 104.52 ± 1.64 (for MMF) to 1515.79 ± 75.86 (for SMF) µmol TE/g dry extract using a calibration curve of trolox (*r*^2^ = 0.9995). Spearman correlation was run to determine the direction and magnitude of the relationship between the GAE and TAC of all extracts. Interestingly, there was a very strong, positive correlation between GAE and TAC (*r*_s_ = 0.929, *n* = 16, *p* < 0.010).

### 3.3. DPPH Radical Scavenging Activity

Table 2 shows that only six from the twelve crude extracts and fractions of *D. indum* fruit parts had IC_50_ values within the tested concentration range (3.91–500.00 μg/mL). None of the mesocarp crude extracts and fractions reached 50% inhibition of DPPH radicals despite there being concentration-dependent DPPH scavenging activity as shown by mesocarp dichloromethane fraction (MDF) and mesocarp hexane fraction (MHF). The IC_50_ values of various crude extracts and fractions varied in the range of 31.71 ± 0.88 (for SMF) to 497.97 ± 6.43 (for exocarp hexane fraction, EHF) µg/mL and maximum percentage of inhibition varied in the range of 27.27 ± 1.08% (for MHF) to 93.11 ± 0.22% (for SMF). Spearman correlation analysis showed that there was a negative correlation between the IC_50_ values of DPPH radical scavenging assay and GAE values of the tested extracts (*r*_s_ = −0.587, *n* = 6, *p* < 0.050). 

A total of twenty-six (26) subfractions were obtained from the fractionation of exocarp dichloromethane fraction (EDF) using column chromatography. Subfractions Di-6, Di-9, Di-11, Di-17, Di-21, Di-22, Di-23, Di-24, Di-25, and Di-26 were observed to possess clearly different TLC antioxidant bioautograms after visualization with 0.4 mM DPPH in methanol (Figure S1). Hence, DPPH radical scavenging activity of these ten EDF subfractions were determined where all selected subfractions presented concentration-dependent DPPH radical scavenging activities. However, only five subfractions, labelled as Di-21, Di-22, Di-24, Di-25, and Di-26 exhibited appreciable antioxidant activity with IC_50_ values of 83.73 ± 0.92, 87.96 ± 1.02, 53.42 ± 1.61, 37.66 ± 0.71, and 69.82 ± 1.28 µg/mL, respectively. Comparison with the IC_50_ value of EDF (260.82 ± 1.31 µg/mL) clearly signified that the five subfractions possessed stronger DPPH radical scavenging activities and thus were considered as antioxidant-enriched EDF subfractions.

### 3.4. Linoleic Acid Peroxidation Inhibition

Table 2 demonstrates that despite the percentage of linoleic acid peroxidation inhibition was significantly lower (*p* < 0.050) than quercetin, both EDF and EHF exhibited the highest percentage of inhibition (51.08 ± 0.84 and 51.46 ± 0.62 µg/mL, respectively) between all samples for the tested concentration range (0.98–125.00 µg/mL). Spearman correlation indicated a very weak but not statistically significant negative correlation (*r*_s_ = −0.197, *n* = 8, *p* > 0.050) between the maximum percentage of linoleic acid peroxidation inhibition and maximum percentage of DPPH radical scavenging of the various *D. indum* crude extracts and fractions.

### 3.5. GC-MS of SMF, EDF and EDF Subfractions

**Figure 1:**
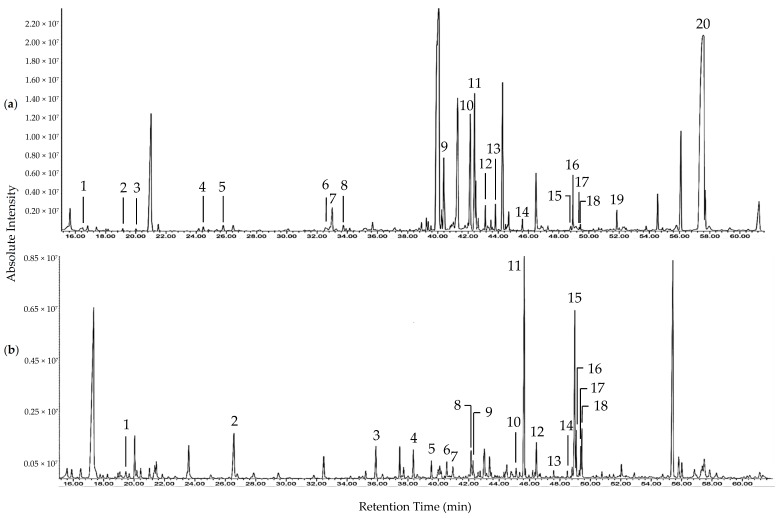
Total ion current (TIC) chromatograms of the trimethylsilyl (TMS)-derivatized *D. indum* seed methanol fraction (SMF) (**a**) and exocarp dichloromethane fraction (EDF) (**b**). Peak numbers refer to metabolites listed in Table 3 and Table 4.

Based on the assays employed, SMF and EDF were considered as fractions with prominent antioxidant activities. The difference in the metabolites of both fractions were investigated using GC-MS after derivatization using MSTFA. The total ion current (TIC) chromatograms of SMF and EDF are shown in Figure 1 and the metabolites identified in both fractions are listed in Table 3 and Table 4. The mass spectra data of the metabolites identified in SMF and EDF are listed in Table S2 [24,25,26,27,28,29,30,31,32,33,34,35,36]. Phenolics, fatty acids, and dicarboxylic acids were detected in both fractions. The total area percentage of phenolics in the TIC chromatogram of EDF was 53 times more than that of SMF. On the other hand, SMF contained amino acids, saccharides, polyol, and sesquiterpene, which were not detected in EDF.

A total of nine phenolics have been identified in EDF, namely vanillic acid, syringic acid, ferulic acid, isoferulic acid, sinapic acid, vanillin, syringic aldehyde, *p*-hydroxybenzaldehyde, and coniferyl aldehyde. Numerous studies correlated the in vitro antioxidant activities of plant extracts with their phenolic contents. GC-MS of five antioxidant-enriched subfractions of EDF added another four phenolics: *p*-hydroxybenzoic acid, homovanillic acid, *p*-coumaric acid, and sinapic aldehyde, to the list of phenolics detected in EDF. The distribution of phenolic antioxidants in subfractions Di-21, Di-22, Di-24, Di-25, Di-26, and EDF is shown in Table 5.

The total area percentage of phenolic antioxidants in the subfractions were lower than EDF, in the following decreasing order: EDF > Di-21 > Di-25 > Di-22 > Di-24 > Di-26. EDF contained the highest number of phenolic antioxidants (9 phenolic antioxidants), followed by Di-22 (8), Di-24 (8), Di-25 (8), Di-21 (4), and Di-26 (3). Vanillin was the major phenolic in EDF. Syringic acid and vanillic acid presented as the major phenolic in subfractions Di-21 and Di-22, respectively, while sinapic acid was the major phenolic in subfractions Di-24, Di-25, and Di-26.

## 4. Discussion

The antioxidant potential of *D. indum* fruit was evaluated by measuring the capabilities of various extracts and fractions of the different fruit parts in reducing phosphomolybdic-phosphotungstic acid reagents, neocuproine, 2,2-diphenyl-picrylhydrazyl (DPPH), and inhibiting linoleic acid peroxidation. The gallic acid equivalence (GAE) for reducing phosphomolybdic-phosphotungstic acid reagents was the highest in the DCM fractions for the exocarp (EDF) and mesocarp (MDF), while for the seed, the highest GAE was measured in the methanol fraction (SMF). The GAE of SMF was found to be the highest when compared with other local underutilized fruits of Malaysia, namely *Baccaurea angulata* [3], *Canarium odontophyllum* [37], and *Sandoricum macropodum* [38] in other studies. It can be suggested from this result that most antioxidants in the exocarp and mesocarp of *D. indum* fruit are semipolar in nature while in the seed, most of the antioxidants are polar. Low GAE values in hexane fractions might be due to the higher concentration of non-polar constituents such as fatty acids. This study also showed a strong, positive correlation (*r*_s_ = 0.929, *n* = 16, *p* < 0.010) between GAE and TAC values, which is in line with a previous study that proved the good correlation (*r* = 0.966) in herbal teas [39]. Both the GAE and TAC values were determined using different assays of a similar underlying antioxidant mechanism, which measure the reduction of the oxidation number of the transition metal ions by antioxidants achieved via electron transfer [40].

The strong neocuproine reducing activity, which was interpreted as the high total antioxidant capacity (TAC) of SMF, can be attributed to the presence of non-phenolic metabolites, particularly the amino acids that also showed neocuproine reducing activity in another study [41]. The following order was ranked for the DPPH radical scavenging activity of the exocarp and seed: SMF > SCM > ECM > EDF > EMF > EHF. However, this sequence was not exactly replicated in their GAE by which the following order was found: SMF > EDF > ECM > EMF > SCM > EHF. The difference in GAE and DPPH radical scavenging activities can be attributed to the difference in the accessibility of various phenolic antioxidants in the different extracts to the unpaired electron of the divalent nitrogen atom of the DPPH radical. Steric accessibility is a major determinant for redox reactions with DPPH, where small antioxidants that have better access to the radical site than their larger counterparts tend to have higher DPPH-reducing power [42]. This finding corroborates previous studies that reported a negative relationship between the IC_50_ values of DPPH radical scavenging and GAE [43,44,45].

Lipid peroxidation involves the formation and propagation of hydroperoxide radicals, which decompose to form byproducts such as malondialdehyde (MDA), ketones, alcohols, and hydrocarbons that can interact with sulfhydryl and amine groups in proteins, causing damage to vital proteins. The distinctive feature of this lipid peroxidation assay lies in the medium used, which is a linoleic acid emulsion, whereas in the GAE, TAC, and DPPH assays, polar media such as water, methanol, and ethanol were used. Moreover, the assay conforms to the antioxidant polar paradox hypothesis, which states that lipid-soluble antioxidants are most effective in emulsions and membranes [46]. It is interesting to note that SMF, which exhibited excellent positive results for all previous assays showed weak linoleic acid peroxidation inhibitory activity with the maximum percentage of inhibition (at 250 µg/mL) below 50%. This finding may be related to the dominance of hydrophilic antioxidants in SMF. The antioxidant activity of compounds in emulsions is attributed to the hydrophilic–lipophilic balance of their polar and hydrocarbon moieties where more lipophilic antioxidants are present in the lipid phase and at the oil-water interface at which interactions between hydroperoxides and prooxidants occur [47].

The strong DPPH radical scavenging activity of SMF can be attributed to the presence of the amino acids detected, namely proline, serine, threonine, pyroglutamic acid, phenylalanine, and glutamic acid. Amino acids were found to exert antioxidant activities by donating protons to electron-deficient radicals [48]. The percentage of amino acids in SMF was four times more than that of the detected phenolics and was higher in the seed than in the exocarp. *D. indum* is a plant in the family Fabaceae, which is a well-known plant family that produces seeds with a high content of proteins and saccharides and are consumed as nutritive food worldwide [49]. The difference in metabolite compositions between EDF and SMF is largely due to the higher percentage of phenolics in EDF (11.48%) than SMF (0.21%). The nine phenolics in EDF can be categorized into two main groups: phenolic acids and phenolic aldehydes. Previous studies have shown that the more the number of hydroxyl groups at the benzene ring, the stronger the DPPH radical scavenging activities of the phenolics and the conjugated carbon skeleton plays an important role in the antioxidant activities of the phenolics. The strength of the in vitro DPPH radical scavenging activities of the phenolics can be ranked in the following decreasing order: hydroxycinnamic acid derivatives > hydroxybenzoic acid derivatives > phenolic aldehydes [50].

Varying percentages of different phenolics have been found in the selected EDF subfractions. Vanillic acid and sinapic acid were consistently detected in EDF and all the selected subfractions, where sub-fraction Di-21 had the highest percentage of vanillic acid (2.07%) while the highest percentage of sinapic acid (3.48%) was found in sub-fraction Di-25. Higher percentages of vanillic acid in EDF, subfractions Di-21 and Di-22 could be related to their weaker DPPH radical scavenging activities than the subfractions with stronger activity, namely subfractions Di-24, Di-25, and Di-26. This finding was parallel to a study that observed an antagonistic effect of vanillic acid when combined with gallic acid, protocatechuic acid, and chlorogenic acid [51]. The stronger DPPH radical scavenging activities of the more polar EDF subfractions viz. Di-24, Di-25, and Di-26 can be related to their higher percentage of hydroxycinnamic acid derivatives (ferulic acid, sinapic acid, and *p*-coumaric acid). Hydroxycinnamic acid derivatives are more potent antioxidants than hydroxybenzoic acid derivatives due to the presence of the additional carbon–carbon double bond next to the benzene ring, thus extending the conjugated π orbital system. This configuration, in turn, stabilizes the resulting phenoxy radicals by resonance and enhances their antioxidant activities [50,52].

## 5. Conclusions

In summary, this study revealed the antioxidant potential of *D. indum* fruit exerted by various extracts and fractions from the different fruit parts. The fruit contains phenolics, amino acids, saccharides, fatty acids, sesquiterpene, polyols, and dicarboxylic acids that collectively contribute to its good antioxidant properties. A combination of fractionation by column chromatography, TLC bioautography, and GC-MS facilitated the identification of thirteen phenolic antioxidants in the exocarp of *D. indum* fruit for the first time. In a nutshell, *D. indum* fruit is a good natural antioxidant source that has great potential for further research and development for pharmaceutical, nutraceutical, and cosmeceutical applications.

## Figures and Tables

**Table 1:** Gallic acid equivalence (GAE) and total antioxidant capacity (TAC) of crude extracts and fractions of *D. indum* fruit.

Extract/Fraction	GAE (µmol GAE/g Dry Extract)	TAC (µmol TE/g Dry Extract)
**Exocarp (E)**		
EHF	95.50 ± 1.57 *	177.00 ± 13.51 *
EDF	439.44 ± 5.73 *	451.48 ± 37.83 *
EMF	258.05 ± 7.85 *	280.77 ± 2.27 *
ECM	316.77 ± 8.35 *	439.39 ± 6.26 *
**Mesocarp (M)**		
MHF	104.06 ± 5.48 *	185.38 ± 9.16 *
MDF	380.54 ± 1.99 *	549.52 ± 27.76 *
MMF	92.97 ± 0.99 *	104.52 ± 1.64 *
MCM	101.56 ± 1.22 *	114.63 ± 1.20 *
**Seed (S)**		
SHF	113.09 ± 1.77 *	259.84 ± 18.63 *
SDF	181.68 ± 1.97 *	336.20 ± 19.93 *
SMF	1405.41 ± 17.96 **	1515.79 ± 75.86 **
SCM	169.38 ± 4.05 *	222.72 ± 16.03 *

HF: hexane fraction; DF: dichloromethane fraction; MF: methanol fraction; CM: crude methanol extract. * Indicates the values in the same column are significantly different (*p* < 0.050) in comparison with SMF (marked **) as measured by one-way analysis of variance (ANOVA) of unequal variance (Welch’s ANOVA) with Games Howell post hoc test.

**Table 2:** 2,2-Diphenyl-picrylhydrazyl (DPPH) radical scavenging and linoleic acid peroxidation inhibition activities of *D. indum* fruit.

Extract/Fraction/ Standard	DPPH Radical Scavenging		Linoleic Acid Inhibition	
	% at 500 µg/mL	IC_50_ (µg/mL)	% at 125 µg/mL	IC_50_ (µg/mL)
**Exocarp (E)**				
EHF	50.13 ± 0.61 *	497.97 ± 6.43 *	51.46 ± 0.62 *	103.26 ± 2.75 *
EDF	74.75 ± 0.70 *	260.82 ± 1.31 *	51.08 ± 0.84 *	121.43 ± 2.97 *
EMF	60.52 ± 0.34 *	415.78 ± 4.48 *	18.83 ± 2.12 *	NA
ECM	92.30 ± 0.08 *	127.63 ± 2.48 *	14.46 ± 0.33 *	NA
**Mesocarp (M)**				
MHF	27.27 ± 1.29 *	NA	33.66 ± 1.19 *	NA
MDF	37.98 ± 0.75 *	NA	11.78 ± 2.34 *	NA
MMF	NA	NA	NA	NA
MCM	NA	NA	NA	NA
**Seed (S)**				
SHF	NA	NA	NA	NA
SDF	NA	NA	17.80 ± 1.78 *	NA
SMF	93.11 ± 0.22	31.71 ± 0.88 *	23.42 ± 1.01 *	NA
SCM	90.99 ± 0.03 *	99.95 ± 0.98 *	20.79 ± 1.43 *	NA
**Standard**				
QUE	94.70 ± 0.02 **	2.40 ± 0.03 **	69.58 ± 0.03 **	44.69 ± 0.17 **

NA: not active; HF: hexane fraction; DF: DCM fraction; MF: methanol fraction; CM: crude methanol extract; QUE: quercetin. * Indicates that the values in the same column are significantly different (*p* < 0.050) in comparison with QUE (marked **) as measured by Welch’s ANOVA with Games Howell post hoc test.

**Table 3:** Metabolites identified in the *D. indum* seed methanol fraction (SMF) through Gas Chromatography-Mass Spectrometry (GC-MS) analysis.

Peak No.	RT (min)	Tentative Metabolite	Similarity Index	M^+^	Molecular Formula	Area (%)
1	16.49	Proline	93	115.06	C_5_H_9_NO_2_	0.09
2	19.14	Serine	91	105.04	C_3_H_7_NO_3_	0.08
3	20.01	Threonine	91	119.06	C_4_H_9_NO_3_	0.06
4	24.47	Malic acid	99	134.02	C_4_H_6_O_5_	0.17
5	25.80	Pyroglutamic acid	95	129.04	C_5_H_7_NO_3_	0.27
6	32.57	Phenylalanine	91	165.08	C_9_H_11_NO_2_	0.16
7	32.86	Glutamic acid	98	147.05	C_5_H_9_NO_4_	0.19
8	33.93	Tartaric acid	94	150.02	C_4_H_6_O_6_	0.07
9	40.39	β-d-Galactofuranose	91	180.06	C_6_H_12_O_6_	2.43
10	42.16	β-d-Glucopyranose	94	180.06	C_6_H_12_O_6_	3.89
11	42.44	d-glucose	91	180.06	C_6_H_12_O_6_	3.96
12	43.14	α-Cyperone	95	218.17	C_15_H_22_O	0.55
13	43.82	*myo*-Inositol	95	180.06	C_6_H_12_O_6_	0.55
14	45.61	Palmitic acid	99	256.24	C_16_H_32_O_2_	0.25
15	48.81	Linoelaidic acid	99	280.24	C_18_H_32_O_2_	0.15
16	48.93	Oleic acid	99	282.26	C_18_H_34_O_2_	0.38
17	49.36	Sinapic acid	99	224.07	C_11_H_12_O_5_	0.07
18	49.44	Stearic acid	99	284.27	C_18_H_36_O_2_	0.12
19	52.25	δ-Tocopherol	99	402.35	C_27_H_46_O_2_	0.14
20	57.55	Sucrose	95	342.12	C_12_H_22_O_11_	18.86
					Total	32.44

RT = Retention Time, C = Carbon, H = Hydrogen, O = Oxygen, N = Nitrogen, M^+^ = molecular ion, *m/z*.

**Table 4:** Metabolites identified in the *D. indum* exocarp dichloromethane (DCM) fraction (EDF) through GC-MS analysis.

Peak No.	RT (min)	Tentative Metabolite	Similarity Index	M^+^	Molecular Formula	Area (%)
1	19.46	*p*-Hydroxybenzaldehyde	97	122.04	C_7_H_6_O_2_	0.45
2	26.57	Vanillin	98	152.05	C_8_H_8_O_3_	3.48
3	35.91	Syringic aldehyde	96	182.06	C_9_H_10_O_4_	1.61
4	38.36	Vanillic acid	96	168.04	C_9_H_10_O_4_	1.26
5	39.55	Azelaic acid	90	188.11	C_9_H_16_O_4_	0.67
6	40.57	Coniferyl aldehyde	94	178.06	C_10_H_10_O_3_	0.67
7	40.97	Myristic acid	99	228.21	C_14_H_28_O_2_	0.50
8	42.16	Syringic acid	99	198.05	C_9_H_10_O_5_	1.24
9	42.62	Ferulic acid	96	194.06	C_10_H_10_O_4_	0.30
10	45.13	Palmitelaidic acid	99	254.23	C_16_H_30_O_2_	0.45
11	45.66	Palmitic acid	99	256.24	C_16_H_32_O_2_	9.71
12	46.46	Isoferulic acid	95	196.06	C_10_H_10_O_4_	1.50
13	47.59	Margaric acid	98	270.26	C_17_H_34_O_2_	0.24
14	48.81	Linoelaidic acid	99	280.24	C_18_H_32_O_2_	0.37
15	48.98	Oleic acid	99	282.26	C_18_H_34_O_2_	7.17
16	49.08	*cis*-Vaccenic acid	99	282.26	C_18_H_34_O_2_	1.41
17	49.37	Sinapic acid	99	224.07	C_11_H_12_O_5_	0.97
18	49.45	Stearic acid	99	284.27	C_18_H_36_O_2_	1.50
					Total	33.50

RT = Retention Time, C = Carbon, H = Hydrogen, O = Oxygen, N = Nitrogen, M^+^ = molecular ion, *m/z*.

**Table 5:** Area percentage (%) of phenolics in exocarp DCM fraction (EDF) and subfractions through GC-MS analysis.

Phenolics	Exocarp DCM Fraction (EDF)	EDF Subfractions				
		Di-21	Di-22	Di-24	Di-25	Di-26
**Phenolic aldehydes**						
*p*-Hydroxybenzaldehyde	0.45	ND	ND	0.02	ND	ND
Vanillin	3.48	ND	0.46	0.23	0.16	ND
Syringic aldehyde	1.61	ND	0.26	0.31	0.3	ND
Coniferyl aldehyde	0.67	ND	ND	0.25	0.11	ND
Sinapic aldehyde *	ND	ND	0.22	0.10	ND	ND
**Phenolic acids**						
Vanillic acid	1.26	2.07	1.30	0.73	0.39	0.45
Syringic acid	1.24	5.14	0.69	0.64	ND	ND
*p*-Hydroxybenzoic acid *	ND	ND	0.05	ND	ND	ND
Homovanillic acid *	ND	2.15	0.23	ND	0.01	ND
Ferulic acid	0.30	ND	ND	ND	1.20	0.44
Isoferulic acid	1.50	ND	ND	ND	ND	ND
Sinapic acid	0.97	0.98	0.17	0.89	3.48	2.15
*p*-Coumaric acid *	ND	ND	ND	ND	0.11	ND
Total	11.48	10.34	3.38	3.17	5.76	3.04

ND: not detected. * Indicates the phenolic was detected only in subfractions.

## Supplementary Materials

The following are available online at http://www.mdpi.com/2076-3921/7/11/154/s1, Table S1: Condition parameters for GC optimization, Table S2: Mass spectra data of metabolites identified in SMF and EDF, Figure S1: TLC antioxidant bioautograms of EDF subfractions (Di-1–Di-26) sprayed with 0.4 mM DPPH in methanol.

## References

[1] Halliwell B. (2007). Biochemistry of oxidative stress. Biochem. Soc. Trans..

[2] Kongkachuichai R., Charoensiri R., Yakoh K., Kringkasemsee A., Insung P. (2015). Nutrients value and antioxidant content of indigenous vegetables from Southern Thailand. Food Chem..

[3] Ahmed I.A., Mikail M.A., Ibrahim M., Hazali N., Rasad M.S.B.A., Ghani R.A., Wahab R.A., Arief S.J., Yahya M.N.A. (2015). Antioxidant activity and phenolic profile of various morphological parts of underutilised *Baccaurea angulata* fruit. Food Chem..

[4] Donno D., Cerutti A.K., Mellano M.G., Prgomet Z., Beccaro G.L. (2016). Serviceberry, a berry fruit with growing interest of industry: Physicochemical and quali-quantitative health-related compound characterisation. J. Funct. Food..

[5] Wang X., Zhang C., Peng Y., Zhang H., Wang Z., Gao Y., Liu Y., Zhang H. (2018). Chemical constituents, antioxidant and gastrointestinal transit accelerating activities of dried fruit of *Crataegus dahurica*. Food Chem..

[6] Kee M.E., Khoo H.E., Sia C.M., Yim H.S. (2015). Fractionation of potent antioxidative components from langsat (*Lansium domesticum*) peel. Pertanika J. Trop. Agric. Sci..

[7] Zefang L., Zhao Z., Hongmei W., Zhiqin Z., Jie Y. (2016). Phenolic composition and antioxidant capacities of Chinese local pummelo cultivars’ peel. Hort. Plant J..

[8] Wongnarat C., Srihanam P. (2017). Phytochemical and antioxidant activity in seeds and pulp of grape cultivated in Thailand. Orient. J. Chem..

[9] Tounkara F., Bashari M., Le G.-W., Shi Y.-H. (2014). Antioxidant activities of roselle (*Hibiscus sabdariffa* L.) seed protein hydrolysate and its derived peptide fractions. Int. J. Food Prop..

[10] Procházková D., Boušová I., Wilhelmová N. (2011). Antioxidant and prooxidant properties of flavonoids. Fitoterapia.

[11] Marcuse R. (1960). Antioxidative effect of amino-acids. Nature.

[12] Milow P., Malek S.B., Edo J., Ong H.C. (2014). Malaysian species of plants with edible fruits or seeds and their valuation. Int. J. Fruit Sci..

[13] Kamarudin M.S., Latiff A., Turner I.M. (2013). Taxonomic realignment of Malaysian vascular plants in Burkill’s monumental dictionary. Malayan Nat. J..

[14] Janick J., Paull R.E. (2008). Fabaceae/Leguminosae. The Encyclopedia of Fruit and Nuts.

[15] Lasekan O., See N.S. (2015). Key volatile aroma compounds of three black velvet tamarind (*Dialium*) fruit species. Food Chem..

[16] Tanjung E., Thalib I., Suhartono E. (2014). Evaluation of antioxidant activity of some selected tropical fruits in South Kalimantan, Indonesia. J. Trop. Life Sci..

[17] Bamikole A.O., Ibidun O.O., Ibitayo O.A., Bolaji A.O., Idowu O.I., Damilola B.B., Abimbola F., Olabisi O.T., Joseph A.O., Funmilayo A. (2018). Evaluation of antioxidant potentials of different solvent-fractions of *Dialium indum* (African black velvet tamarind) fruit pulp—In vitro. Potravinarstvo Slovak J. Food Sci..

[18] Ismail M., Bagalkotkar G., Iqbal S., Adamu H.A. (2012). Anticancer properties and phenolic contents of sequentially prepared extracts from different parts of selected medicinal plants indigenous to Malaysia. Molecules.

[19] Apak R., Güçlü K., Özyürek M., Bektaşoǧlu B., Bener M., Armstrong D. (2008). Cupric ion reducing antioxidant capacity assay for food antioxidants: Vitamins, polyphenolics and flavonoids in food extracts. Advanced Protocols in Oxidative Stress I. Methods in Molecular Biology.

[20] Li W.J., Cheng X.L., Liu J., Lin R.C., Wang G.L., Du S.S., Liu Z.L. (2012). Phenolic compounds and antioxidant activities of *Liriope muscari*. Molecules.

[21] Kapila S., Vibha P.R.S. (2006). Antioxidative and hypocholesterolemic effect of *Lactobacillus casei* ssp *casei* (biodefensive properties of lactobacilli). Indian J. Med. Sci..

[22] Alimi H., Hfaiedh N., Bouoni Z., Sakly M., Rhouma K.B. (2011). Evaluation of antioxidant and antiulcerogenic activities of *Opuntia ficus indica* f. *inermis* flowers extract in rats. Environ. Toxicol. Pharmacol..

[23] Derivatization Reagents for Selective Response and Detection in Complex Matrices. https://www.sigmaaldrich.com/content/dam/sigmaaldrich/migrationresource4/Derivatization%20Rgts%20brochure.pdf.

[24] Khallouki F., Haubner R., Erben G., Ulrich C.M., Owen R.W. (2012). Phytochemical composition and antioxidant capacity of various botanical parts of the fruits of *Prunus* × *domestica* L. from the Lorraine region of Europe. Food Chem..

[25] Esmaeili N., Ebrahimzadeh H., Abdi K., Safarian S. (2011). Determination of some phenolic compounds in *Crocus sativus* L. corms and its antioxidant activities study. Pharmacogn. Mag..

[26] Martin J.G.P., Porto E., Corrêa C.B., De Alencar S.M., Da Gloria E.M., Cabral I.S.R., De Aquino L.M. (2012). Antimicrobial potential and chemical composition of agro-industrial wastes. J. Nat. Prod..

[27] Katona Z., Sass P., Molnár-Perl I. (1999). Simultaneous determination of sugars, sugar alcohols, acids and amino acids in apricots by gas chromatography–mass spectrometry. J. Chromatogr. A.

[28] Füzfai Z., Katona Z.F., Kovács E., Molnár-Perl I. (2004). Simultaneous identification and quantification of the sugar, sugar alcohol and carboxylic acid contents of sour cherry, apple and ber fruits, as their trimethylsilyl derivatives, by gas chromatography−mass spectrometry. J. Agric. Food Chem..

[29] Roessner U., Wagner C., Kopka J., Trethewey R.N., Willmitzer L. (2000). Simultaneous analysis of metabolites in potato tuber by gas chromatography-mass spectrometry. Plant J..

[30] Plessi M., Bertelli D., Miglietta F. (2006). Extraction and identification by GC-MS of phenolic acids in traditional balsamic vinegar from Modena. J. Food Compost. Anal..

[31] Ng L.-K., Lafontaine P., Harnois J. (2000). Gas chromatographic–mass spectrometric analysis of acids and phenols in distilled alcohol beverages. J. Chromatogr. A..

[32] Guo J., Shi Y., Xu C., Zhong R., Zhang F., Zhang T., Niu B., Wang J. (2016). Quantification of plasma myo-inositol using gas chromatography–mass spectrometry. Clin. Chim. Acta.

[33] Kilani S., Ledauphin J., Bouhlel I., Sghaier M.B., Boubaker J., Skandrani I., Mosrati R., Ghedira K., Barillier D., Chekir-Ghedira L. (2008). Comparative study of *Cyperus rotundus* essential oil by a modified GC/MS analysis method. Evaluation of its antioxidant, cytotoxic and apoptotic effects. Chem. Biodivers..

[34] NIST Chemistry WebBook. https://webbook.nist.gov/.

[35] Zhang K., Zuo Y. (2004). GC-MS determination of flavonoids and phenolic and benzoic acids in human plasma after consumption of cranberry juice. J. Agric. Food Chem..

[36] Lytovchenko A., Beleggia R., Schauer N., Isaacson T., Leuendorf J.E., Hellmann H., Rose J.K.C., Fernie A.R. (2009). Application of GC-MS for the detection of lipophilic compounds in diverse plant tissues. Plant Methods.

[37] Shakirin F.H., Prasad K.N., Ismail A., Yuon L.C., Azlan A. (2010). Antioxidant capacity of underutilized Malaysian *Canarium odontophyllum* (dabai) Miq. fruit. J. Food Compost. Anal..

[38] Ikram E.H.K., Eng K.H., Jalil A.M.M., Ismail A., Idris S., Azlan A., Nazri H.S.M., Diton N.A.M., Mokhtar R.A.M. (2009). Antioxidant capacity and total phenolic content of Malaysian underutilized fruits. J. Food Compost. Anal..

[39] Apak R., Güçlü K., Ozyürek M., Karademir S.E., Erçağ E. (2006). The cupric ion reducing antioxidant capacity and polyphenolic content of some herbal teas. Int. J. Food Sci. Nutr..

[40] Huang D., Boxin O.U., Prior R.L. (2005). The chemistry behind antioxidant capacity assays. J. Agric. Food Chem..

[41] Imer F., Aldemir E., Kiliç H., Sonmezoǧlu I., Apak R. (2008). The protective effect of amino acids on the copper(II)-catalyzed autoxidation of ascorbic acid. J. Food Drug Anal..

[42] Pyrzynska K., Pękal A. (2013). Application of free radical diphenylpicrylhydrazyl (DPPH) to estimate the antioxidant capacity of food samples. Anal. Methods.

[43] Clarke G., Ting K., Wiart C., Fry J. (2013). High correlation of 2,2-diphenyl-1-picrylhydrazyl (DPPH) radical scavenging, ferric reducing activity potential and total phenolics content indicates redundancy in use of all three assays to screen for antioxidant activity of extracts of plants from the Malaysian rainforest. Antioxidants.

[44] Javadi N., Abas F., Mediani A., Hamid A.A., Khatib A., Simoh S., Shaari K. (2015). Effect of storage time on metabolite profile and alpha-glucosidase inhibitory activity of *Cosmos caudatus* leaves—GCMS based metabolomics approach. J. Food Drug Anal..

[45] Lai H., Lim Y. (2011). Evaluation of antioxidant activities of the methanolic extracts of selected ferns in Malaysia. Int. J. Environ. Sci. Dev..

[46] Barden L., Barouh N., Villeneuve P., Decker E. (2015). Impact of hydrophobicity on antioxidant efficacy in low-moisture food. J. Agric. Food Chem..

[47] Yehye W.A., Rahman N.A., Alhadi A.A., Khaledi H., Ng S.W., Ariffin A. (2012). Butylated hydroxytoluene analogs: Synthesis and evaluation of their multipotent antioxidant activities. Molecules.

[48] Wang J., Hu S., Nie S., Yu Q., Xie M. (2016). Reviews on mechanisms of in vitro antioxidant activity of polysaccharides. Oxid. Med. Cell. Longev..

[49] Gulewicz P., Martinez-Villaluenga C., Kasprowicz-Potocka M., Frias J. (2014). Non-nutritive compounds in Fabaceae family seeds and the improvement of their nutritional quality by traditional processing—A review. Pol. J. Food Nutr. Sci..

[50] Mathew S., Abraham T.E., Zakaria Z.A. (2015). Reactivity of phenolic compounds towards free radicals under in vitro conditions. J. Food Sci. Technol..

[51] Palafox-Carlos H., Gil-Chávez J., Sotelo-Mundo R.R., Namiesnik J., Gorinstein S., González-Aguilar G.A. (2012). Antioxidant interactions between major phenolic compounds found in “Ataulfo” mango pulp: Chlorogenic, gallic, protocatechuic and vanillic acids. Molecules.

[52] Yamagami C., Akamatsu M., Motohashi N., Hamada S., Tanahashi T. (2005). Quantitative structure-activity relationship studies for antioxidant hydroxybenzalacetones by quantum chemical- and 3-D-QSAR (CoMFA) analyses. Bioorg. Med. Chem. Lett..

