# Supplementary material for: Antioxidant Activities of Dialium indum L. Fruit and Gas Chromatography-Mass Spectrometry (GC-MS) of the Active Fractions
Source: Antioxidants (Basel). 2018 Nov 1;7(11):154. doi: 10.3390/antiox7110154 (PMC6262551; doi:10.3390/antiox7110154)
Supplement: Supplementary file 1 [file antioxidants-07-00154-s001.pdf]

antioxidants

MDPI

Article

Supplementary materials: Antioxidant Activities of *Dialium indum* L. Fruit and Gas Chromatography-Mass Spectrometry (GC-MS) of the Active Fractions

Muhamad Faris Osman [1], Norazian Mohd Hassan [1,*], Alfi Khatib [1] and Siti Marponga Tolos [2]

[1] Department of Pharmaceutical Chemistry, Kulliyyah of Pharmacy, International Islamic University Malaysia, Kuantan 25200, Pahang, Malaysia; farisosman@iium.edu.my (M.F.O.); alfikhatib@iium.edu.my (A.K.)

[2] Department of Computational and Theoretical Sciences, Kulliyyah of Science, International Islamic University Malaysia, Kuantan 25200, Pahang, Malaysia; smtolos@iium.edu.my

* Correspondence: norazianmh@iium.edu.my; Tel.: +60-9-570-4937

Table S1. Condition parameters for GC optimization.

**Condition**	**Initial T (T_0) (°C)**	**Initial t (t_0) (min)**	**Rate to T_1 (°C/min)**	**Final T (T_1) (°C)**	**Final t (t_1) (min)**	**Rate to T_2 (°C/min)**	**Final T (T_2) (°C)**	**Final t (t_2) (min)**
A1	50	6	10	180	34	50	315	5
B1	50	10	5	90	10	5	250	0
C1	50	12	5	90	10	5	250	10
C2	50	12	5	100	10	5	250	10
C3	50	12	5	120	10	5	250	10
C4	50	12	5	120	10	5	300	10
C5	50	12	5	150	10	5	250	10
D1*	100	12	5	140	10	5	250	10
D2	100	12	5	150	10	5	250	10
D3	100	12	5	160	10	5	250	10
D4	100	12	5	170	10	5	250	10
D5	100	12	5	180	10	5	250	10
D6	100	12	5	190	10	5	250	10

* Optimum condition. A condition is considered optimum when the chromatogram shows best peaks separation (no overlapping) and number of phenolics identified with similarity index of 90 and above is the highest.

Table S2. Mass spectra data of metabolites identified in SMF and EDF.

Compound (TMS derivative)	Ions, *m*/*z* (rel. intensity, %)	Ions, *m*/*z* (rel. intensity, %) from literature	Reference
**Amino acids**			
Proline	216(8), 147(9), 143(13), 142(100), 73(29)	216, 186, 142	[27]
Serine	218(62), 205(19), 204(100), 147(17), 73(51)	306, 218, 204	[27]
Threonine	291(41), 219(84), 218(93), 117(44), 73(100)	291	[29]
Pyroglutamic acid	258(13), 157(13), 156(100), 147(16), 73(33)	156, 147, 73	[34]
Phenylalanine	219(20), 218(100), 192(65), 147(17), 73(56)	218	[29]
Glutamic acid	247(21), 246(100), 128(17), 147(17), 73(42)	246	[29]
**Organic acids**			
Tartaric acid	293(19), 292(66), 219(30), 147(62), 73(100)	292	[31]
Malic acid	245(23), 233(37), 147(78), 133(17), 73(100)	335, 233	[27]
Azelaic acid	317(100), 201(49), 126(36), 75(87), 73(99)	317(M^+), 201(30), 129(26), 55(40), 73(100), 43(30)	[26]
**Fatty acids**			
Palmitic acid	313(100), 129(29), 117(55), 75(34), 73(46)	313, 117	[27]
Linoelaidic acid	337(86), 81(45), 75(83), 73(100), 67(47)	337, 81, 75, 73, 67	
Oleic acid	339(100), 129(57), 117(62), 75(62), 73(87)	339, 129, 117	[27]
Stearic acid	341(100), 132(30), 117(55), 75(34), 73(47)	341, 129, 117	[27]
Myristic acid	285(100), 129(32), 117(59), 75(41), 73(55)	285, 129, 117	[27]
Palmitelaidic acid	311(100), 129(61), 117(57), 75(68), 73(78)	311, 129, 117, 75, 73	[34]
Margaric acid	327(100), 129(36), 117(59), 75(42), 73(65)	327, 129, 117	[27]
cis-Vaccenic acid	339(100), 129(47), 117(43), 75(50), 73(55)	339, 129, 117, 75, 73	[34]
**Tocopherol**			
δ-Tocopherol	474(100), 209(24), 208(21), 73(28)	474	[36]

Table S2. (*Continued*).

Compound (TMS derivative)	Ions, *m*/*z* (rel. intensity, %)	Ions, *m*/*z* (rel. intensity, %) from literature	Reference
**Polyol**			
myo-Inositol	246(16), 218(20), 217(100), 147(22), 73(39)	217, 147, 73	[32]
**Sesquiterpene**			
α-Cyperone	280(15), 220(9), 219(20), 218(100), 73(31)	218	[33]
**Saccharides**			
Sucrose	362(31), 361(100), 217(34), 147(17), 73(47)	361	[28]
β-D-Galactofuranose	218(20), 217(100), 191(17), 147(18), 73(39)	217, 191, 147, 73	[34]
β-D-Glucopyranose	205(19), 204(100), 191(39), 147(20), 73(40)	217, 204, 191, 147, 73	[34]
D-glucose	217(27), 204(100), 191(50), 147(21), 73(44)	307, 204	[28]
**Phenolics**			
Sinapic acid	368(100), 338(79), 312(45), 131(84), 73(82)	368, 353, 338, 249, 207, 161, 73	[35]
p-Hydroxybenzaldehyde	194(77), 179(100), 151(61), 75(24), 73(47)	194(77), 179(100), 151(62)	[24]
Vanillin	224(31), 209(46), 195(16), 194(100), 193(51)	224(33), 209(48), 194(100)	[24]
Syringic aldehyde	254(30), 239(43), 225(17), 224(100), 223(28),	254(32), 239(46), 224(100)	[24]
Vanillic acid	312(63), 297(100), 267(64), 253(40) 223(46)	312(68), 297(100), 282(29), 267(65), 253(41), 223(43)	[24]
Coniferyl aldehyde	250(88), 220(100), 219(78), 192(51), 73(41)	250(94), 235(38), 220(100), 192(51)	[24]
Syringic acid	342(76), 327(100), 312(71), 297(55), 73(45)	342(80), 327(100), 312(70), 297(54), 253(33)	[24]
Ferulic acid	338(56), 323(40), 308(29), 223(100), 73(52)	338, 323, 308, 293, 249, 219	[25]
Isoferulic acid	338(100), 323(58), 308(48), 223(61), 73(60)	338, 323, 249, 161	[30]

References:

24. Khallouki, F.; Haubner, R.; Erben, G.; Ulrich, C.M.; Owen, R.W. Phytochemical composition and antioxidant capacity of various botanical parts of the fruits of *Prunus × domestica* L. from the Lorraine region of Europe. *Food Chem*. **2012**, *133*, 697–706, doi:10.1016/j.foodchem.2012.01.071.

25. Esmaeili, N.; Ebrahimzadeh, H.; Abdi, K.; Safarian, S. Determination of some phenolic compounds in *Crocus sativus* L. corms and its antioxidant activities study. *Pharmacogn. Mag.* **2011**, *7*, 74–80, doi:10.4103/0973-1296.75906.

26. Martin, J.G.P.; Porto, E.; Corrêa, C.B.; De Alencar, S.M.; Da Gloria, E.M.; Cabral, I.S.R.; De Aquino, L.M. Antimicrobial potential and chemical composition of agro-industrial wastes. *J. Nat. Prod.* **2012**, *5*, 27–36.
27. Katona, Z.; Sass, P.; Molnár-Perl, I. Simultaneous determination of sugars, sugar alcohols, acids and amino acids in apricots by gas chromatography–mass spectrometry. *J. Chromatogr. A* **1999**, *847*, 91–102, doi:10.1016/s0021-9673(99)00333-7.
28. Füzfai, Z.; Katona, Z.F.; Kovács, E.; Molnár-Perl, I. Simultaneous identification and quantification of the sugar, sugar alcohol and carboxylic acid contents of sour cherry, apple and ber fruits, as their trimethylsilyl derivatives, by gas chromatography-mass spectrometry. *J. Agric. Food Chem*. **2004**, *52*, 7444–7452, doi:10.1021/jf040118p.
29. Roessner, U.; Wagner, C.; Kopka, J.; Trethewey, R.N.; Willmitzer, L. Simultaneous analysis of metabolites in potato tuber by gas chromatography-mass spectrometry. *Plant J*. **2000**, *23*, 131–142. doi:10.1046/j.1365-313x.2000.00774.x.
30. Plessi, M.; Bertelli, D.; Miglietta, F. Extraction and identification by GC-MS of phenolic acids in traditional balsamic vinegar from Modena. *J. Food Compost. Anal.* **2006**, *19*, 49–54, doi:10.1016/j.jfca.2004.10.008.
31. Ng, L.-K.; Lafontaine, P.; Harnois, J. Gas chromatographic–mass spectrometric analysis of acids and phenols in distilled alcohol beverages. *J. Chromatogr. A.* **2000**, *873*, 29–38, doi:10.1016/s0021-9673(99)01100-0.
32. Guo, J.; Shi, Y.; Xu, C.; Zhong, R.; Zhang, F.; Zhang, T.; Niu, B.; Wang, J. Quantification of plasma myo-inositol using gas chromatography–mass spectrometry. *Clin. Chim. Acta* **2016**, *460*, 88–92, doi:10.1016/j.cca.2016.06.022.
33. Kilani, S.; Ledauphin, J.; Bouhlel, I.; Sghaier, M.B.; Boubaker, J.; Skandrani, I.; Mosrati, R.; Ghedira, K.; Barillier, D.; Chekir-Ghedira, L. Comparative study of *Cyperus rotundus* essential oil by a modified GC/MS analysis method. Evaluation of its antioxidant, cytotoxic and apoptotic effects. *Chem. Biodivers*. **2008**, *5*, 729–742, doi:10.1002/cbdv.200890069.
34. NIST Chemistry WebBook. Available online: https://webbook.nist.gov/ (accessed on 22 September 2018).
35. Zhang, K.; Zuo, Y. GC-MS determination of flavonoids and phenolic and benzoic acids in human plasma after consumption of cranberry juice. *J. Agric. Food Chem*. **2004**, *52*, 222–227, doi:10.1021/jf035073r.
36. Lytovchenko, A.; Beleggia, R.; Schauer, N.; Isaacson, T.; Leuendorf, J.E.; Hellmann, H.; Rose, J.K.C.; Fernie, A.R. Application of GC-MS for the detection of lipophilic compounds in diverse plant tissues. *Plant Methods* **2009**, *5*, 4, doi:10.1186/1746-4811-5-4.

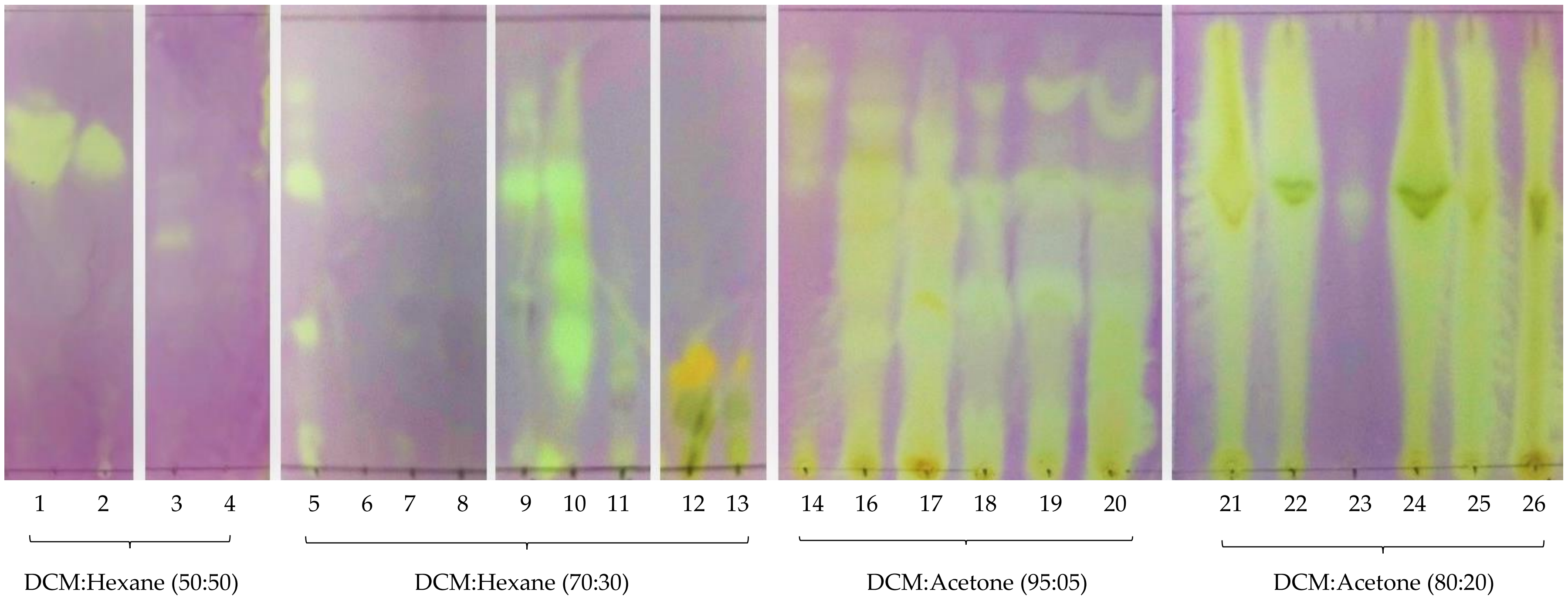

Figure S1. TLC antioxidant bioautograms of EDF subfractions (Di-1-Di-26) sprayed with 0.4 mM DPPH in methanol. Chromatographic conditions: aluminium TLC plate, silica gel 60 Å of 200 μm thickness, coated with fluorescent indicator F_{254}. Solvent systems are as labelled.
